# Transarterial Coil Embolization for Type II Endoleak After Endovascular Aneurysm Repair (EVAR)

**DOI:** 10.7759/cureus.68882

**Published:** 2024-09-07

**Authors:** Ioakeim Giagtzidis, Ioakeim Papoutsis, Theodoros Dimkas, Christos Diamantidis, Georgios Avgeris, Christos Karkos, Konstantinos Papazoglou

**Affiliations:** 1 5th Surgical Department, Hippokrateio General Hospital, Aristotle University of Thessaloniki, Thessaloniki, GRC

**Keywords:** abdominal aortic aneurysm, coiling, endoleak type ii, endovascular, evar

## Abstract

Background

Endovascular aneurysm repair (EVAR) has evolved into treatment of choice for infrarenal abdominal aortic aneurysms (AAA). Type II endoleaks, although frequently benign, can lead to sac enlargement and rupture. Management of these endoleaks by endovascular means can be quite challenging and may require complex techniques and assistance of interventional radiologists, not always available in all vascular units. This is a single-center study of management of type II endoleaks with transarterial coil embolization performed by vascular surgeons and with minimum requirements regarding the necessary equipment.

Methods

From 2017 to 2022, 13 patients with type II endoleak were treated. Local anaesthesia and transfemoral or transbrachial approach was used. The superficial mesenteric artery (SMA) was catheterized and through the Riolan arch, coiling of the inferior mesenteric artery and/or the sac aneurysm was performed.

Results

The mean time period between the primary EVAR procedure and the transarterial intervention for the endoleak was 3.9 years. Primary technical success was achieved in 11 (84.6%) patients, while secondary technical success was 12 (92.3%). In the mean follow-up period, which was 2.6 years, the endoleak was treated successfully in 11 (84.6%) patients.

Conclusions

Transarterial coil embolization of type II endoleaks is a minimal low-cost procedure, with small percentage of complications, high technical and treatment success rates. It could be considered as a first-line treatment of unresolvable type II endoleaks, minimizing the need for open repair.

## Introduction

More than 75% of elective repairs of abdominal aortic aneurysms (AAAs) today are treated with the endovascular aneurysm repair (EVAR) technique [1]. Lower periprocedural mortality, complications and length of hospital stay as well as early and fast rehabilitation that is associated with EVAR can justify the rapid and universal implementation of this treatment option over open repair [2]. However long-term complications are still more frequent in EVAR patients, with endoleak occurring in up to one-third of the cases in the long term [3]. Specifically, endoleaks from collateral vessels (type II) are the most common type. EVAR patients may develop endoleak type II in the early follow-up period which can resolve with no intervention (18%) or it may be persistent (5%) while another 11% of them can present at a later time frame, during follow-up [4]. Half of the late or persistent type II endoleaks result in sac growth, and although the risk of rupture is low (<1%), there seems to be a high re-intervention rate of up to 50% at two years [5]. They are also divided into type IIa endoleak when there is a single vessel involved, with a “to-and-fro” flow, and type IIb when multiple collateral vessels preserve the endoleak [4].

Various treatment options have been described for the treatment of type II endoleak. The endovascular approach involves embolization of the aneurysm sac and lumbar or inferior mesenteric artery (IMA) with a variety of materials through different access points [6]. Surgical repair is usually reserved when endovascular embolization has failed, and treatment options include laparoscopic or open ligation of the “feeding” vessels and/or aneurysmatectomy with or without graft preservation.

The aim of this study is to present the collective experience of patients with type II endoleak after EVAR treated with transarterial coil embolization performed by vascular surgeons from a single center.

## Materials and methods

A retrospective review of all consecutive patients with type II endoleak, treated with transarterial coil embolization in a vascular surgery unit based at a university hospital was performed, gathering data from 2017 to 2022. During this five-year period, a total of 13 patients with type II endoleak were identified. Medical records were reviewed to document gender, age, comorbidities, past medical history, risk factors and use of anticoagulant and/or antiplatelet drugs. The time period between the primary EVAR procedure and the endovascular treatment of the type II endoleak was recorded for each patient. In addition, we recorded whether the endoleak was type IIa or type IIb. Indication for the treatment of the type II endoleak was a persistent type II endoleak resulting in aneurysm sac expansion, usually of 1cm or more compared to the preoperative size. All data were collected from the patients' medical records after consent was acquired. Follow-up data were obtained either from clinical visits or via phone call. To present the results comprehensively, absolute and relative frequencies (percentages %) were calculated using basic descriptive statistics.

**Figure 1:**
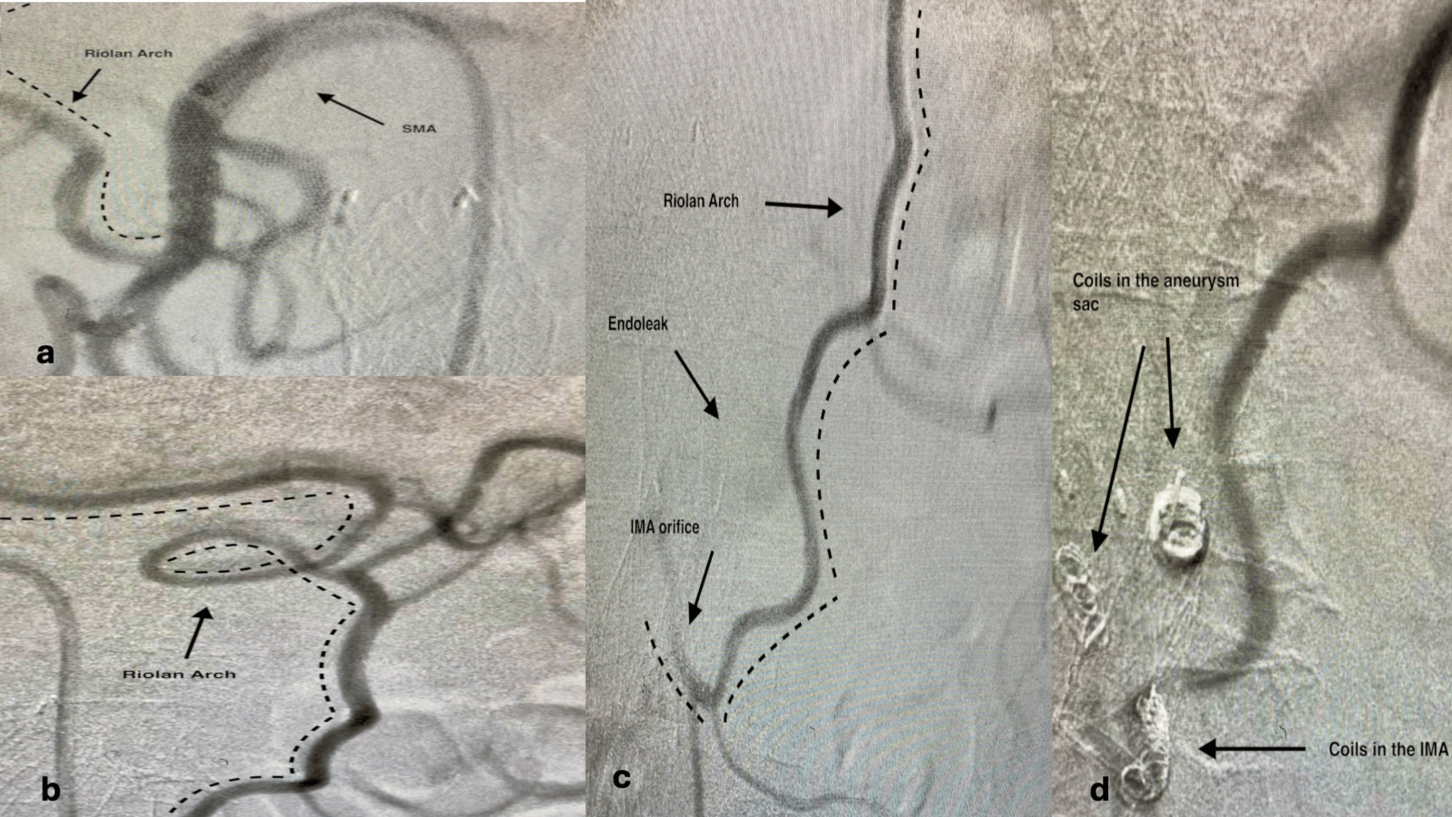
Riolan arch catheterization Original images from the study a: Catheterization of the SMA and identification of the Riolan arch branch, b: Advancement through the Riolan arch, c: Visualization of the IMA and the endoleak, d: Coil deployment into the aneurysm sac and the IMA SAM: superficial mesenteric artery; IMA: inferior mesenteric artery

**Figure 2:**
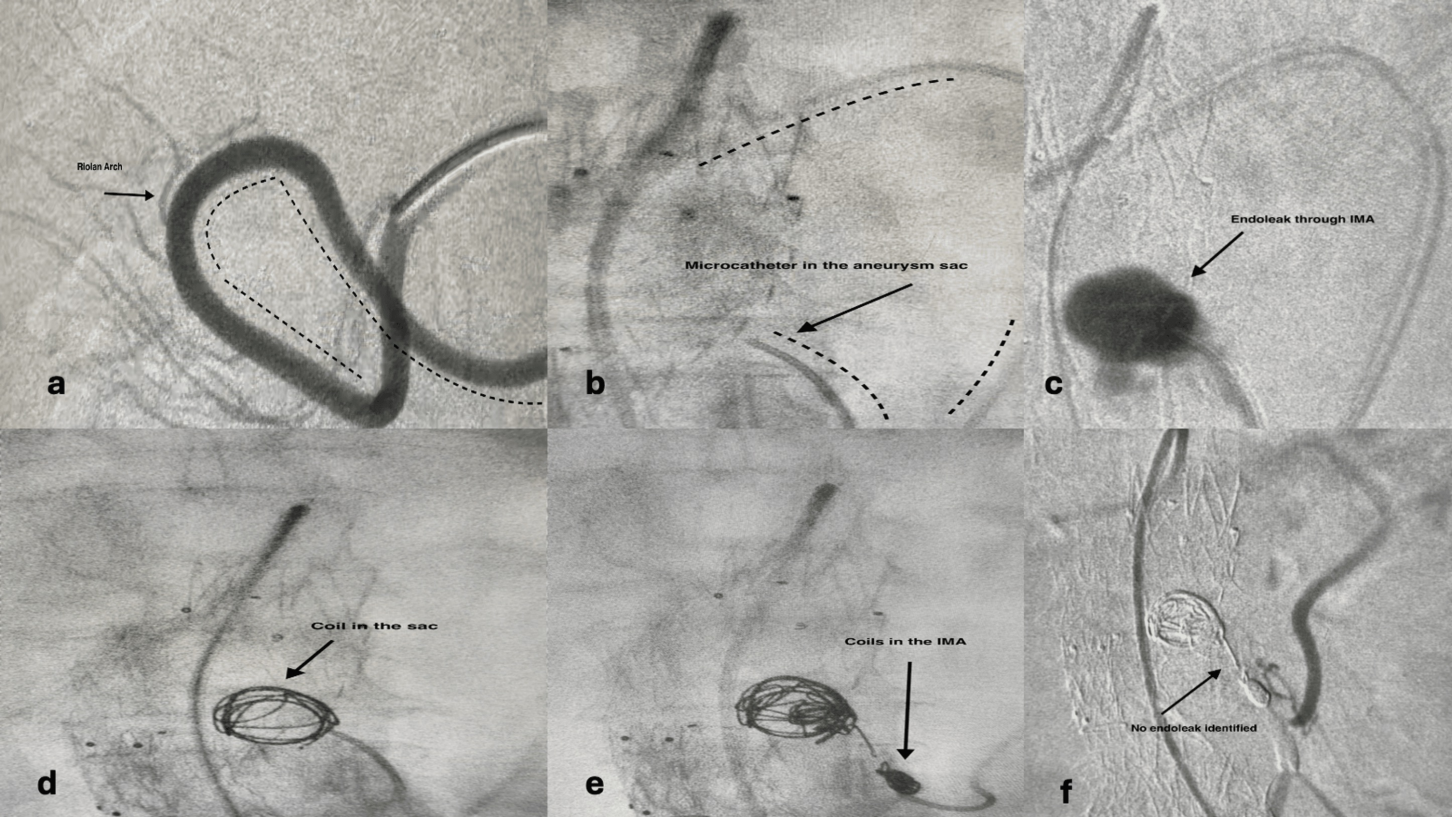
Riolan arch catheterization with microcatheter Original images from the study a: Catheterization of the SMA and the Riolan arch, b: Advancement of the microcatheter into the aneurysm sac, c: Visualization of the IMA and the endoleak, d: Coil deployment into the aneurysm sac, e: Coil deployment to the IMA, f: Final angiography. SAM: superficial mesenteric artery; IMA: inferior mesenteric artery

The procedure was performed under local anesthesia and percutaneous common femoral artery puncture. A 6F sheath, 45cm or 65cm long (Arrow; Teleflex, Wayne, PA, USA or Destination, Terumo, Tokyo, Japan) was inserted. 5.000 units of heparin were administered intravenously. The superficial mesenteric artery (SMA) was selectively catheterized with a suitable 5F catheter (usually a Cobra or a Simmons) and, after identifying angiographically the connecting branch with the IMA (Figure 1), the catheter was advanced in the IMA in retrograde fashion all the way back into the aneurysm sac. In cases of difficulty, a microcatheter (Renegade; Boston Scientific, Alpharetta, GA, USA) was inserted through the first catheter to facilitate advancement and gain access to the sac (Figure 2). A variety of coils and microcoils (Interelock-35 fibered IDC detachable embolization coils; Interlock-18, fibered IDC detachable embolization coils, Boston Scientific, or AZUR CX peripheral coil system, Terumo Interventional Systems) were used to fill the sac or occlude the IMA stem and branches.

Care was taken not to compromise the vascular supply of the left colon and the sigmoid colon. When a transfemoral access was not technically feasible, a left transbrachial or transaxillary approach was attempted instead. In cases of persistent type IIb endoleaks, catherization of the target lumbar arteries was performed transfemorally in similar fashion and techniques after obtaining access through the internal iliac artery system.

Patients were normally discharged the following day. Post-procedure, follow-up included serial Computed Tomography Angiography (CTA). The usual protocol included a CTA at six months, one year and annually thereafter. 

## Results

Of the 13 patients who underwent the operation, 12 were male, the mean age was 74.5 (range 58-88) years. Patient demographics and comorbidities are presented in Table 1.

**Table 1:** Patients demographics and comorbidities.

Gender	Ν(%)
Male	12(92.3%)
Female	1(7.6 %)
Comorbidities	
Smoking	13(100%)
Hypertension	12(92.3%)
Chronic renal insufficiency	9(69.2%)
Dyslipidemia	9(69.2%)
Cardiac insufficiency	2(15.3%)
Atrial fibrillation	4(30.7%)
Coronary disease	4(30.7%)
Diabetes mellitus	5(38.4%)
Cerebrovascular disease	1(7.6%)
History of Cancer	1(7.6%)

In terms of clinical presentation, the majority were asymptomatic and two were complaining of vague abdominal or back pain. The mean time period between the primary EVAR procedure and the transarterial intervention for the endoleak was 3.9 years (range two months to seven years). Of the 13 patients, 11 (84.6%) had late endoleaks, i.e not present in the initial follow-up imaging, whereas two (15.3%) were early and persistent type II endoleaks. In addition, 11 (84.6%) endoleaks presented with a patent dominant IMA, one (7.6%) with only lumbar arteries and one (7.6%) with both IMA and lumbar arteries patent.

**Figure 3:**
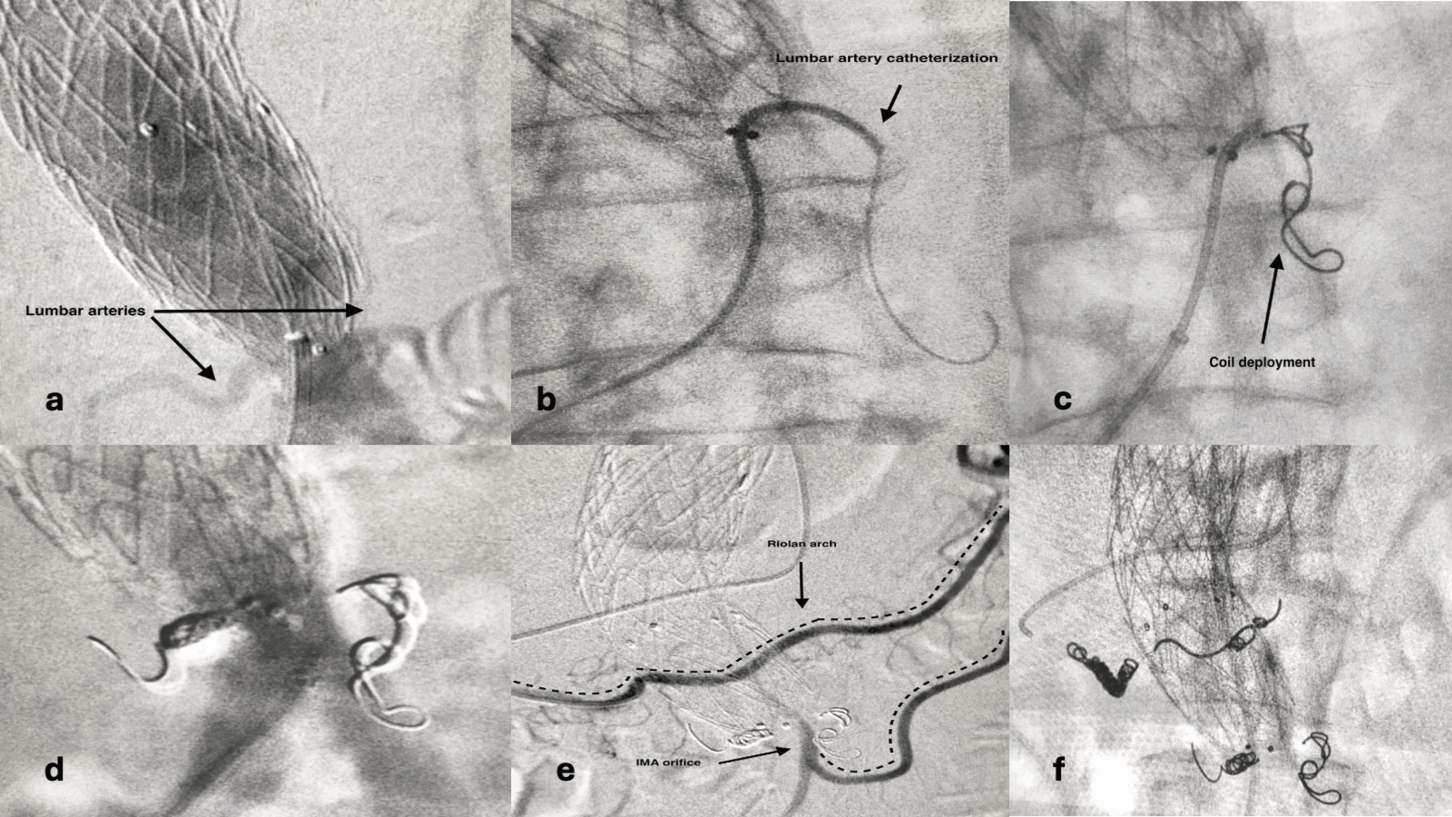
Coiling of both lumbar and Riolan arch in a patient Original images from the study a: Lumbar arteries visualization, b: Lumbar artery catheterization, c: Coil deployment in lumbar arteries, d: Final angiography of thrombosed lumbar arteries, e: Catheterization of the SMA, Riolan arch and visualization of the IMA, f: Coil deployment in the Riolan arch SAM: superficial mesenteric artery; IMA: inferior mesenteric artery

All patients were operated under local anesthesia. All but one procedures were performed transfemorally. One patient had a transfemoral attempt which was unsuccessful because of failure to achieve stable catheterization of the SMA. The procedure was repeated two days later via a transaxillary approach which was successful. In another patient, the catheterization of the SMA was successful, but it was not possible to catheterize the arterial branch which was supplying the arc of Riolan. A localized rupture occurred and, as a result, the procedure was abandoned with no adverse sequela. A further procedure for transarterial embolization was attempted 18 months later, but again was unsuccessful, because it was not possible to visualize the responsible anastomotic arterial branch with the middle colic artery. In one patient with a type IIb endoleak, coil embolization of two lumbar arteries was performed (Figure 3A-3E). After six months, a type IIA endoleak was identified and a second transarterial intervention was performed for coil embolization of the IMA (Figure 3F).

In all other patients the endoleak was treated successfully through the primary approach mentioned above. There were no deaths or no peri-procedural adverse events. Specifically, there were no puncture-related local hematomas, pseudoaneurysms, or limb ischemia and no cases of bowel or buttock ischemia. The mean number of coils used per operation was 3.1 (range 2-5). Based on the above, the technical success rate of the transarterial embolization procedure was 11 out of 13 (84.6%) after the primary procedure and 12 out of 13 patients (92.3%) after a second attempt to embolize.

The follow-up spanned a mean time period of 2.6 years (range 7 months to 5.2 years). With regard to the one patient whose both embolization attempts were unsuccessful, due to further aneurysm growth, he eventually had open surgical exploration. Specifically, he underwent laparotomy, ligation of the IMA and lumbar arteries, opening of the sac without cross-clamping, thrombus evacuation and sac plication and re-suturing. Despite the good initial result, six months later he presented with fever, malaise and evidence of sac-enteric fistula. This required a further exploratory laparotomy, detachment and resection of the involved bowel segment and reanastomosis, debridement of the anterior wall of the sac, preservation of the endograft and closure of the sac defect with omentum. Despite a stormy four-week hospital stay, he recovered well and is in satisfactory condition three months later. The patient, who had combined IIb and IIa endoleaks, continued to have sac growth two years after the transarterial embolization procedures. He eventually underwent open conversion with endograft preservation, ligation of the IMA and lumbar arteries and sac plication. He is well three years later. 

With regard to the remaining patients, after one year of monitoring, one patient was lost to follow-up and two patients died from causes not related to their aneurysm. No patient developed a new type II endoleak during the follow-up. Two patients developed a type Ia endoleak and underwent a successful endovascular intervention with proximal aortic extensions.

## Discussion

Type II endoleaks, which are originating from collateral vessels of the aneurysm sac, were originally described by White et al. [7] and remain along with the other types of endoleak,,one of the most important drawback of EVAR. They occur when there is retrograde flow into the aneurysm sac originating mainly from lumbar arteries or the IMA and rarely from an accessory renal, gonadal or median sacral arteries [8].

They are notably the most frequent with an incidence rating from 5% to 35% [9]. They can be divided as type IIa when there is a single collateral patent with a “to and fro” flow in the aneurysm sac, and type IIb when multiple collaterals are participating in maintaining a flow circuit [10]. A type II endoleak may be early, when is present within the first month after EVAR, persistent, when it lasts more than six months, or late when it is diagnosed 12 months postoperatively [10]. Factors that are associated with presence of type II endoleaks are the number of patent lumbar arteries and patency of IMA preoperatively, their diameter (lumbar arteries >1.9mm, IMA >2.5mm), sac thrombus and possibly anticoagulation [11-13].

There are many studies that advocate that most type II endoleaks are innocuous and a significant proportion of them self-resolve (59.8% - 75%) [3,14]. Additionally, it is stated that even persistent or untreated type II endoleaks did not result in aneurysm rupture and increased aneurysm-related mortality [6,15]. On the contrary, there is also robust evidence that persistent or late type II endoleaks can lead to sac growth and rupture or be responsible for a high re-intervention rate and conversion to open [16,17].

As a solution, a pre-EVAR aneurysm sac embolization has been suggested in order to minimize the incidence of type II endoleak [18,19]. A meta-analysis confirmed that pre-EVAR IMA embolization dropped type II endoleaks to a rate of 19.9% compared to 41.4% incidence with standard EVAR [20]. However, the authors concluded that since treatment for this type of endoleak is needed in less than 20% of the cases and given that aneurysm rupture risk is around 0.9%, preemptive IMA embolization is not supported [20].

Considering the contradictory data of how benign type II endoleaks are, recent guidelines recommend that sac expansion of 1cm³ after EVAR is a significant growth threshold. If type II endoleak is suspected and all other endoleak types are ruled out, re-intervention should be considered primarily by endovascular means [4].

Endovascular treatment options for type II endoleaks include transarterial embolization of the IMA or the aneurysm sac through the SMA and the arc of Riolan or the marginal artery [21]. Endoleaks from lumbar arteries can be accessed either directly or through retrograde cannulation of hypogastric and iliolumbar arteries [22], while transcaval approach is rarely performed and only if the endoleak is in close proximity to the inferior vena cava [23]. In order to treat the endoleak, it is important to advance all the way into the aneurysm sac and eliminate all inflow and outflow vessels which is not always possible due to long and tortuous collateral pathways. In that case, several authors suggest the use of steerable guiding sheath as Destino (Oscor Inc., Palm Harbor, FL, USA) or Morph (BioCardia Inc., San Carlos, CA, USA) [8].

Embolization materials include coils, which are most widely used, n-butyl-cyanocrylate glue and Onyx (ethylene-vinyl alcohol copolymer, EVOH) liquid embolic system (Medtronic, Dublin, Ireland) [24]. One disadvantage of the above materials is that they produce a beam hardening artifact in follow-up CT scans which can make diagnosing persistent or new endoleaks difficult. Finally, direct sac access can be achieved through direct translumbar puncture under CT guidance usually followed by glue embolization [25].

A retrospective study with 84 patients comparing transarterial with translumbar embolization came up with a success rate of 78% and 72% respectively with no statistically significant difference [26]. Additionally, a similar study, comparing 23 patients undergoing 35 embolizations with the above minimal invasive techniques, found no difference in persistence type II endoleak and procedure complications but shorter fluoroscopy and procedure time with the translumbar approach [27]. On the contrary, a systematic review published in 2013, showed that translumbar approach may have higher success rate and fewer complications, but overall endovascular treatment is successful in 60-80% of the cases [6]. In this study series the primary success rate was 84.6% and the secondary success rate of 92.3% with no new type II endoleaks in the successfully embolized patients over a mean follow-up period of 2.6 years. These overall different results from several studies verify that type II endoleak is a complicated and multifactorial finding following EVAR, with only consensus that close surveillance and observation is warranted [28].

This study has several limitations. It is a single-center retrospective study with a small number of patients included but with an adequate follow-up period. Furthermore, there is no comparison of other therapeutic strategies, but this is beyond the scope of this paper. 

## Conclusions

Endoleak remains the Achilles’ heel of EVAR technique. Especially for type II, although it is quite common, it will not always be a threat for aneurysm sac expansion and rupture. Ideally it would be useful to be able to predict which of the type II endoleaks will self-thrombose, and which of them will lead to sac expansion and require treatment. Since there is now a large number of patients with type II endoleak, well-designed trials are required to identify confounding factors, to establish predictive models and to compare different treatment approaches, different devices and embolents.

The above-described endovascular techniques can be concluded with relatively common devices and materials that are available in most vascular units with low complication and high success rates. Although as always, a learning curve is required, mastering access of IMA and lumbar arteries is feasible and fulfilling for any vascular/endovascular surgeon, especially when translumbar approach which requires an active interventional radiology department is not always accessible. 
